# Single-molecule studies reveal reciprocating of WRN helicase core along ssDNA during DNA unwinding

**DOI:** 10.1038/srep43954

**Published:** 2017-03-07

**Authors:** Wen-Qiang Wu, Xi-Miao Hou, Bo Zhang, Philippe Fossé, Brigitte René, Olivier Mauffret, Ming Li, Shuo-Xing Dou, Xu-Guang Xi

**Affiliations:** 1 College of Life Sciences, Northwest A&F University, Yangling 712100, China; 2 LBPA, IDA, ENS Cachan, CNRS, Université Paris-Saclay, Cachan F-94235, France; 3 Beijing National Laboratory for Condensed Matter Physics and CAS Key Laboratory of Soft Matter Physics, Institute of Physics, Chinese Academy of Sciences, Beijing 100190, China; 4 School of Physical Sciences, University of Chinese Academy of Sciences, Beijing 100049, China

## Abstract

Werner syndrome is caused by mutations in the WRN gene encoding WRN helicase. A knowledge of WRN helicase’s DNA unwinding mechanism *in vitro* is helpful for predicting its behaviors *in vivo*, and then understanding their biological functions. In the present study, for deeply understanding the DNA unwinding mechanism of WRN, we comprehensively characterized the DNA unwinding properties of chicken WRN helicase core in details, by taking advantages of single-molecule fluorescence resonance energy transfer (smFRET) method. We showed that WRN exhibits repetitive DNA unwinding and translocation behaviors on different DNA structures, including forked, overhanging and G-quadruplex-containing DNAs with an apparently limited unwinding processivity. It was further revealed that the repetitive behaviors were caused by reciprocating of WRN along the same ssDNA, rather than by complete dissociation from and rebinding to substrates or by strand switching. The present study sheds new light on the mechanism for WRN functioning.

Helicases are ubiquitous enzymes moving directionally along nucleic acids by coupling the chemical energy from NTP to the separation of two annealed nucleic acid strands^1^. They are found in all known organisms and involved in practically all aspects of nucleic acid metabolism, including replication, recombination, transcription, translation, repair, chromosome segregation and telomere maintenance^1^,^2^,^3^. Helicases in the RecQ family, named after the 3′–5′ DNA helicase RecQ from *Escherichia coli*, have been highly conserved during evolution from bacteria to human and play essential roles in maintaining genome stability owing to their versatility^3^,^4^,^5^. In human, there are five RecQ homologs, and deficiencies in three of them, BLM/RecQ2, WRN/RecQ3 and RTS/RecQ4, give rise, respectively, to Bloom^6^, Werner^7^ and Rothmund-Thomson^8^ syndromes that are characterized by genomic instability and cancer predisposition^4^.

Werner syndrome is an autosomal recessive disorder, and patients exhibit features of premature aging including cataracts, osteoporosis, atherosclerosis and cancer^9^. WRN helicase contains four conservative domains and is the only RecQ helicase having both 3′–5′ DNA helicase activity (with the helicase domain, the RecQ C-terminal (RQC), and the helicase and RNaseD C-terminal (HRDC)) and 3′–5′ exonuclease activity (with the exonuclease domain), which, however, need not to function concertedly^10^,^11^. In some model organisms, the helicase and the exonuclease domains homologous to human WRN are even encoded by different genes^12^,^13^,^14^. Human and mice lacking the helicase function of WRN exhibit many phenotypic features of Werner, indicating the helicase activity is a crucial function of the WRN protein^9^. Furthermore, WRN helicase activity is essential for many special processes, such as maintaining fragile site stability^15^, telomere lagging strand synthesis^16^ and enabling Pol δ to synthesize past secondary structures^17^. WRN helicase can nonprocessively unwind less than ~30 bp DNA duplexes in the absence of any auxiliary factors with a 3′ to 5′ polarity^7^,^18^,^19^,^20^, and efficiently unwinds a short forked duplex substrate with 3′- and 5′-ssDNA tail lengths of no less than 10 nucleotides at different protein concentrations^18^. In addition, a WRN helicase fragment has been shown to be nearly as efficient as the full-length WRN^20^. However, because of the difficulty to efficiently purify WRN^21^ and technical limitations, its unwinding mechanism remains obscure in vertebrates.

In recent decades, advancements in single-molecule techniques, such as optical tweezers, magnetic tweezers, single-molecule fluorescence resonance energy transfer (smFRET), laminar flow and DNA curtains, revolutionize our ability to investigate the functions and properties of helicases in real time^22^. Using single-molecule probing techniques, we and other groups have studied BLM helicase core^23^,^24^,^25^,^26^ at the single-molecule level, and revealed some unprecedented details.

In this study, we efficiently expressed and purified chicken WRN helicase core. Using smFRET, we found a novel feature of WRN in DNA unwinding: repetitive movements. The repetitive movements happened on forked, 3′/5′-overhanging, and G4-containing DNA substrates and results in different phenomena. On forked and 3′-overhanging duplex DNA, WRN might be driven backwards along the tracking strand by DNA rezipping when it was binding loosely to this strand, resulting in an apparently limited unwinding processivity. On 5′-overhanging duplex DNA, WRN was anchoring at the ss/dsDNA junction and reeled in the 5′ ssDNA. On G4-containing duplex DNA, when encountering the G4 structure, WRN also made repetitive movements, resulting in repetitive unfolding and refolding of G4. The repetitive movements came neither from its dissociation and rebinding, nor from strand switching, rather, it came from reciprocating of the helicase along the same ssDNA. This novel feature of WRN might keep dsDNA or other DNA secondary structures such as G4 and hairpin being constantly in a disrupted or unfolded state, and thus make contributions to the functioning of other enzymes during transcription, replication, DNA repair and telomere metabolism *in vivo*.

## Results

### WRN exhibited both one-step and repetitive unwinding with a forked DNA

**Figure 1:**
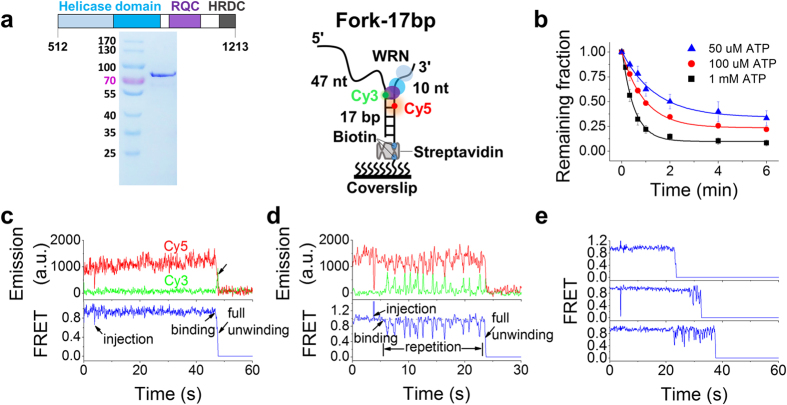
WRN-catalyzed unwinding of Fork-17bp in smFRET assay. (**a**) Analysis of the purified WRN helicase by SDS PAGE (left panel) and structure of the Cy3- and Cy5-labelled substrate Fork-17bp (right panel). DNA is immobilized on the streptavidin-coated coverslip surface through biotin. (**b**) Fractions of DNA molecules remaining on the coverslip surface versus time after addition of 1 nM WRN and different concentrations of ATP (Error bar = s.d.; *n* = 3). Fittings of the data to a single-exponential decay yielded the corresponding unwinding rates at different ATP concentrations (Supplementary Table S2). (**c,d**) Two typical time traces of fluorescence intensities of Cy3 and Cy5 (upper panel) and the corresponding FRET traces (lower panel) at 1 nM WRN and 1 mM ATP. (**c**) One-step full unwinding; (**d**) Repetitive oscillation before full unwinding. Full unwinding is indicated by an arrow. (**e**) Time traces showing the time at which full unwinding occurs lies in a wide range.

Chicken WRN is an ortholog of human WRN and their helicase domains share 75% identity at the amino acid level^27^. In addition, chicken DT40 cells have been shown to be a valuable model system for investigating the cellular functions of BLM and WRN^27^,^28^. To facilitate our research, we expressed and purified *Gallus gallus* WRN helicase core, which includes the helicase, RQC and HRDC domains (Fig. 1a, left panel). For simplicity, we used WRN to represent the WRN helicase core in this work, unless specified otherwise.

To characterize the general unwinding properties of WRN, we designed a substrate to mimic a replication fork or an open telomeric end, named as Fork-17bp (Fig. 1a, right panel), which was labelled with Cy3 (donor) at the ss/dsDNA junction, and Cy5 (acceptor) on the biotinylated strand at the 5^th^ nucleotide from the junction (sequence listed in Supplementary Table S1). The substrate was immobilized to the PEG surface via biotin. Such spaced donor and acceptor can report the unwinding of the duplex sensitively by the FRET signal. After 1 nM WRN was injected into the chamber with ATP at different concentrations to unwind the Fork-17bp substrate under single-molecule conditions, the remaining fraction of DNA, determined by counting the number of remaining Cy5 spots per imaging area^26^, was decreasing with time (Fig. 1b). To confirm the disappearances of Cy5 spots were indeed due to the intrinsic unwinding activity of WRN that led to release of the donor strands, we prepared a single-tailed dsDNA by removing the 3′-tail (named as 47nt-17bp, Supplementary Fig. S1a). In the presence of 1 nM WRN and 1 mM ATP, WRN failed to unfold 47nt-17bp (Supplementary Fig. S1b,c), just as expected because WRN is a strict 3′–5′ helicase^19^.

By inspecting the individual smFRET traces for Fork-17bp with 1 nM WRN and 1 mM ATP, we found there were typically two distinct patterns: one-step full unwinding (Fig. 1c, ~40%) and repetitive oscillation before full unwinding (Fig. 1d, ~60%). Furthermore, even at 10 nM WRN, the ratio was not changed (Supplementary Fig. S2a). In the case of one-step unwinding, once WRN was loaded on and unwound the substrate, the emission of Cy5 decreased to zero, together with the rising and abrupt disappearing of Cy3 emission (Fig. 1c, arrow in upper panel). The simultaneous disappearance of both dyes should not result from photobleaching, because of the extremely low probability for their simultaneous photobleaching. In the case of repetitive oscillation before full unwinding, the FRET signal displayed oscillation or bursting, with each burst characterized by a gradual decrease to ~0.6, followed by an much faster recovery of the FRET signal (Fig. 1d and Supplementary Fig. S2b,c), with an average dwell time of 0.67 s between two successive bursts (Supplementary Fig. S2d). The FRET decrease was attributed to unwinding of the DNA duplex by WRN, and the faster recovery to reannealing of the unwound DNA. The repetitive unwinding events might be caused by complete dissociation from and rebinding to the DNA substrate by different WRN molecules (or complexes), or by repetitive functioning of a single enzyme (or complex). By inspection of a series of data, it was observed that sometimes a long time-interval appeared between two repetitive unwinding events (Supplementary Fig. S2e), which strongly suggests that a single enzyme or complex is responsible for one repetitive unwinding event. To make further confirmation, we pre-incubated 10 nM WRN with Fork-17bp for 5 min and then the unbound proteins were washed away by using 10 volumes of imaging buffer containing 1 mM ATP. Repetitive unwinding event was still observed (Supplementary Fig. S2f). Therefore, we concluded that the FRET bursting phenomenon was resulted from the activity of a single enzyme or complex, which is, very likely, a WRN monomer^29^. From a large amount of data, we also found that the number of bursts in each repetitive unwinding event lies in a wide range (Fig. 1e) and, on average, five bursts occur before the full unwinding of a substrate. In addition, the lowest FRET values in a repetitive unwinding event are ~0.3 (Supplementary Fig. S3a), while full unwinding corresponds to much lower FRET values.

### Repetitive unwinding came from the reciprocating of WRN along ssDNA

**Figure 2:**
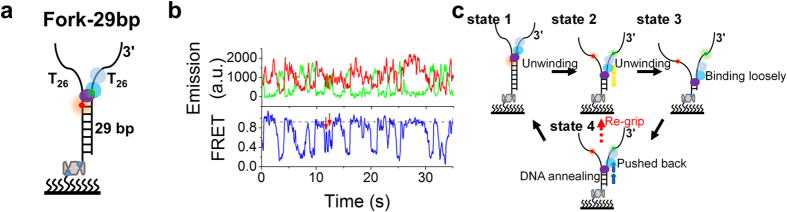
WRN-catalyzed unwinding of Fork-29bp and the proposed model. (**a**) Schematic diagram of substrate Fork-29bp. (**b**) Typical time traces of fluorescence intensities of Cy3 and Cy5 (upper panel) and the corresponding FRET trace (lower panel). The new interaction phases are indicated by red arrows, corresponding to the ‘re-grip’ process in (**c**) (i.e., state 4 to 2). (**c**) Proposed model for WRN-catalyzed unwinding of Fork-29bp. State 1, WRN binding at the fork and starting to unwind the duplex. The two RecA-like domains bind and translocate along the tracking ssDNA while RQC interacts with and melts the DNA duplex at the ss/dsDNA junction. State 2, WRN has unwound some base pairs. State 3, in a certain nucleotide state during DNA unwinding, the two RecA-like domains loosen their strong binding to the tracking ssDNA. State 4, WRN is pushed backwards along the tracking strand by DNA annealing, while RQC remains contacting with the retreating ss/dsDNA junction. WRN can be pushed back until DNA annealing is completed (state 1), or, alternatively, WRN may re-grip the tracking strand and resume unwinding of the partially annealed duplex (state 2).

To further investigate the mechanism for the repetitive unwinding behaviors of WRN, we designed a fork structure with a longer dsDNA stem (Fig. 2a, named as Fork-29bp). Compared with Fork-17bp under the same conditions, both unwinding fraction and rate were decreased with this new substrate (Supplementary Fig. S3b). Furthermore, only repetitive unwinding could sometimes be observed (Fig. 2b), with FRET values lower than 0.3 (Supplementary Fig. S3a). No one-step full unwinding happened, very probably because of the limited processivity of WRN^20^,^30^. In a repetitive unwinding event, most bursts were still characterized by a gradual decrease, followed by a rapid recovery of the FRET signal, similar to that observed with Fork-17bp (Supplementary Fig. S2b–d). Because of the increased FRET change with the new substrate (compare Figs 1d and 2b), details of the bursts could be seen more clearly. Remarkably, a new feature was observed: the oscillating FRET signal might return only to an intermediate value instead of the initial value (Fig. 2b, red arrows, and Supplementary Fig. S4).

In consideration of the similar interactions of RecQ^31^, BLM^32^,^33^, and WRN^34^ with DNA, the gradual FRET decrease in a burst can be easily interpreted as WRN-catalyzed duplex unwinding (Supplementary Fig. S5a–c), in which the two RecA-like domains bind and translocate along the tracking ssDNA while RQC interacts with the DNA duplex at the ss/dsDNA junction. The latter is ATP-independent and strong, whereas the former depends on the nucleotide state and may become weak during an ATP hydrolysis cycle (Supplementary Fig. S5c) just like RecA^35^ and BLM^36^. This was confirmed by equilibrium DNA binding assay for WRN (Supplementary Fig. S6).

For the gradual decrease and rapid increase in a burst of FRET, we have considered the following three possible models (Supplementary Fig. S5). i) Switch and translocation: RQC remains attached to the ss/dsDNA junction, while the two RecA-like domains release the 3′-tail, then switch and translocate along the 5′-tail in the 3′–5′ direction, followed by reannealing of the unwound DNA (Supplementary Fig. S5d). Finally, WRN switches strand again and re-grips the 3′ ssDNA (Supplementary Fig. S5a). ii) Switch and sliding: it is similar to the above model, except that WRN slides back along the 5′-tail in the 3′–5′ direction, as pushed by reannealing of the unwound DNA (Supplementary Fig. S5d’). Finally, WRN switches strand again and re-grips the 3′ ssDNA (Supplementary Fig. S5a). iii) Reciprocating: WRN does not make strand switch but is pushed backwards along the tracking strand in the 5′-3′ direction when the two RecA-like domains weaken their binding to the tracking strand (Supplementary Fig. S5d”). Then, the two RecA-like domains re-strengthen their binding to the 3′ ssDNA again (Supplementary Fig. S5a).

Among the three mechanistic models, WRN should rely on the 5′-tail of the DNA substrate in the first (Switch and translocation) and the second (Switch and sliding) models. Thus if there is no 5′-tail, the FRET signal cannot return back to its initial level. To see if this is true, we removed the 5′-tail of Fork-29bp, yielding a 3′-tailed substrate (29bp-26nt, Supplementary Fig. S7a). With this new substrate and under the same conditions as for Fork-29bp (Fig. 2b), repetitive unwinding still occurred and the FRET signal could still return back to its initial level (Supplementary Fig. S7b). This result indicates clearly that the 5′-tail is not indispensable, thus providing no support for the first two models. Besides this experiment, the two models are also opposed by the following two points. First, RecQ family helicases are typically active ones, with RQC binding and effectively melting dsDNA, thus duplex unwinding is as fast as its translocation along ssDNA^37^. If the Switch and translocation model is true, then in each burst we should observe FRET decreasing and increasing with similar rates, rather than gradual decreasing and abrupt recovering (Supplementary Fig. S2b,c). Second, in the Switch and sliding model, because of the binding polarity of WRN with ssDNA^38^,^39^, WRN should interact with the forked DNA in an intertwined mode (Supplementary Fig. S5d’) and thus is unlikely to slide back quickly along the 5′-tail.

As shown in Fig. 2c, the repetitive unwinding/annealing phenomenon can be well explained by the Reciprocating model (see the legend for more details). In addition, the new feature that the oscillating FRET signal returns to an intermediate value instead of the initial value sometimes (Fig. 2b, red arrows, and Supplementary Fig. S4) can also be explained as resulting from a mode change of WRN from the forced backward movement to unwinding, which occurs as soon as its strong interaction with the tracking strand is resumed^40^,^41^. It is noteworthy that in the Reciprocating model, the binding polarity of WRN with DNA always remains unchanged. Actually, there are many other DNA-interaction proteins, including helicases, that reciprocate along ssDNA with polar loading^40^,^41^,^42^,^43^,^44^,^45^.

### WRN induced looping of the 5′-ssDNA overhang

**Figure 3:**
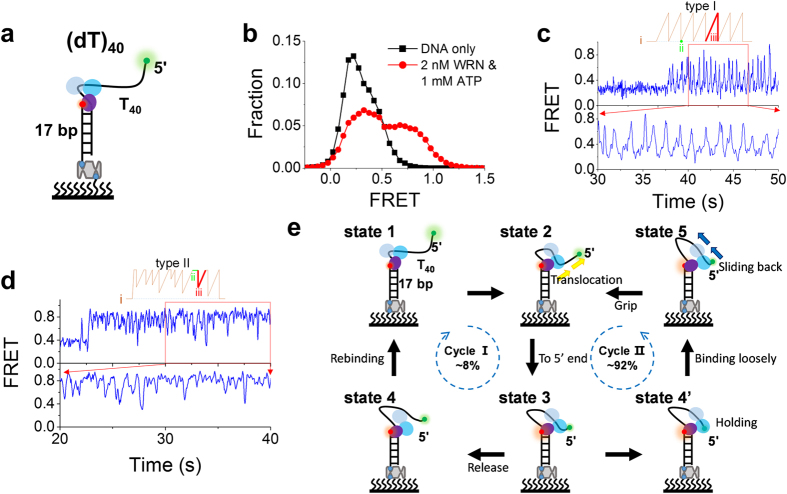
WRN induces looping of the 5′-ssDNA overhang. (**a**) Schematic diagram of substrate (dT)_40_, which contains a duplex DNA with a 40-nt poly(dT) 5′-overhang, labelled with Cy3 at the 5′-tail end and Cy5 at the ss/dsDNA junction. (**b**) FRET histograms obtained before and 20 s after adding 2 nM WRN and 1 mM ATP. (**c,d**) Two typical patterns of FRET changes: (**c**) periodic sawtooth-shaped bursting, (**d**) irregular bursting. (**e**) Our proposed model to explain how WRN induces looping of the 5′-ssDNA overhang (see more details in the text).

In addition to the forked DNA structures studied above, WRN may encounter substrates without a free 3′ ssDNA tail. For example, WRN has been shown to play important roles in telomere lagging-strand replication^16^ and in resolving secondary structures that block DNA polymerases at stalled replication forks^15^ (Supplementary Fig. S8a). Therefore, we used a duplex DNA with a 40-mer poly(dT) 5′-overhang, labelled with Cy3 and Cy5 at the two ends, to simply mimic this structural environment (Fig. 3a, named as (dT)_40_). Before initiation of the reaction, the initial FRET signal was low (Fig. 3b, black square) because of the 40-nt spatial separation of the two dyes. However, 20 s after addition of 2 nM WRN and 1 mM ATP, the low-FRET population obviously decreased, while the high-FRET population increased (Fig. 3b, red circle). There was no such obvious FRET changes when ATP was replaced by the nonhydrolyzable ATP analog, adenyl-5′-yl imidodiphosphate (AMP-PNP) (Supplementary Fig. S9a), indicating that the FRET changes were indeed ATP-dependent. They should be induced by a WRN binding at the ss/dsDNA junction and pulling the 5′-terminal to the junction, similar to previous cases of BLM^26^, Pif1p^46^, and PcrA^47^. Examination of the FRET traces shows the existence of two main patterns: regular sawtooth-shaped bursting (Type I, 33/412 = 8%) (Fig. 3c) and irregular bursting (Type II, 379/412 = 92%) (Fig. 3d). There are three criteria that were used to distinguish between the two types of bursting: i) Type-I smFRET traces display regular sawtooth-shaped bursts while Type-II traces do not; ii) There is no break between two successive bursts in Type-I traces, but frequently there is in Type-II traces; iii) Each burst in Type-I traces begins with a gradual rising phase followed by an abrupt decreasing phase, while that in Type-II traces begin with an abrupt decreasing phase followed by a gradual rising phase (Fig. 3c,d). Interestingly, these two types of bursting events could switch directly from one to another (Supplementary Fig. S9b). The direct switching together with the fact that the bursting events were separated by pauses (Supplementary Fig. S9c,d) indicate that a single enzyme is responsible for a bursting event.

Based on structures of RecQ family helicases^31^,^32^,^33^,^34^, we propose the following mechanism for the above observed phenomena (Fig. 3e). WRN ‘sits’ at the junction through interaction of RQC with the duplex DNA and reels in the ssDNA tail through ssDNA translocation activity of the two RecA-like domains, while extruding an ssDNA loop (Fig. 3e, states 1 and 2). When the two RecA-like domains meet the 5′ terminal of the ssDNA (state 3), further reactions may occur in two different pathways. i) If WRN releases the 5′ terminal (state 4), it will instantaneously rebind the ssDNA tail near the ss/dsDNA junction, completing a reeling-release-rebinding and reeling cycle (Cycle I), just like PcrA^47^ and Pif1p^46^. ii) Alternatively, WRN may hold the 5′ terminal while still hydrolyzing ATP (state 4′). When binding of the two RecA-like domains to the ssDNA becomes weak, the two domains may slide back quickly along the ssDNA (Fig. 3e, state 5). Once the binding becomes strong again (i.e., re-grip), WRN reels in the ssDNA again, completing a reeling-sliding-re-grip and reeling cycle (Cycle II). It should be pointed out that the ‘re-grip’ in the previous ‘reciprocating model’ (Fig. 2c, states 4 to 2) is similar to the ‘re-grip’ in the above Cycle II, and further supporting our interpretation in Fig. 2c. Thus we can also rule out the possibility that the two RecA-like domains release the tracking strand completely in Fig. 2c (states 4 to 2).

**Figure 4:**
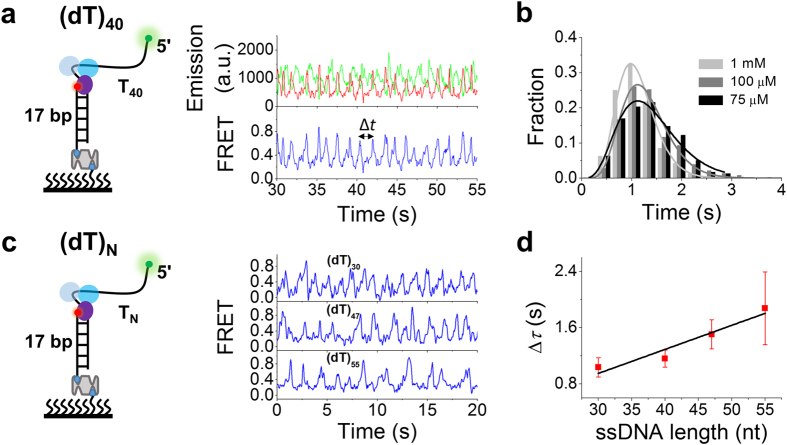
WRN loops 5′ ssDNA periodically. (**a**) Typical time traces of fluorescence intensities of Cy3 and Cy5 and FRET obtained with (dT)_40_. Δ*t* is defined as the time between two successive FRET peaks. (**b**) Histograms of Δ*t* at different ATP concentrations were best fitted by a γ-distribution with time constant Δ*τ* = 1.12, 1.31 and 1.42 s for 1 mM (27 traces), 100 μM (30 traces) and 75 μM ATP (20 traces), respectively. (**c**) Representative time traces of FRET showing repetitive looping of 5′-ssDNA tails with different lengths. (**d**) Time constant Δ*τ* versus 5′-tail length at 1 mM ATP. A WRN translocation rate of 30 ± 6 nt/s was obtained by a linear fit of the data (Error bar = s.e.m.).

Having established that WRN can reel in the 5′ ssDNA, we next further studied the properties of this activity. In Type-I bursting traces such as that shown in Figs 3c and 4a, the periodic FRET bursting is characterized by a repetition interval Δ*t*. Histograms of Δ*t* obtained with different traces can be best fitted by a γ-distribution (Fig. 4b), implying that WRN reels in the 5′-ssDNA tail through multiple steps. Furthermore, the average value of Δ*t* increases with decreasing ATP concentration, which is consistent with the ATP-dependence of ssDNA translocation activity of WRN. We also studied the influence of the 5′-tail length on Δ*t* (Fig. 4c). By a linear fitting of the data (Fig. 4d), we obtained an ssDNA translocation rate of 30 ± 6 nt/s at 1 mM ATP.

**Figure 5:**
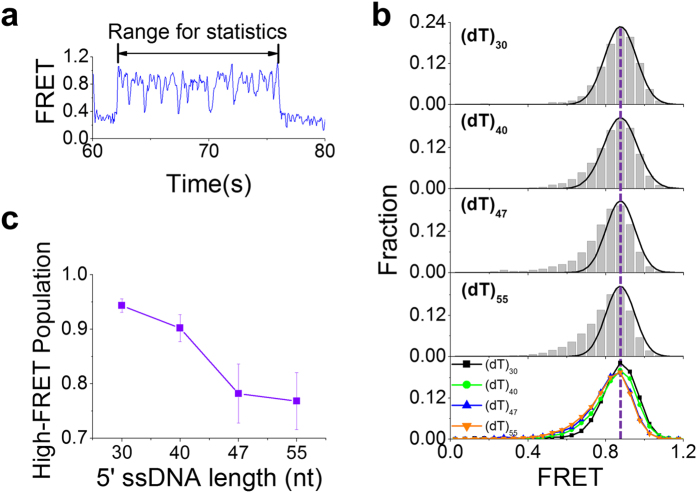
Type-II FRET bursting for DNA substrates with different 5′-tail lengths. (**a**) Representative FRET trace showing type-II bursting. (**b**) Histograms of the bursting FRET signal for different 5′-tail lengths. Solid lines are Gaussian fittings with the same high-FRET peak positions at FRET = 0.87. (**c**) The high-FRET population, obtained from the peak area in (**b**), versus ssDNA length (Error bar = s.e.m.).

In Type-II bursting traces such as that shown in Figs 3d and 5a, the FRET bursts are irregular, we constructed FRET histograms for four different 5′-tail lengths (Fig. 5b). Each histogram is characterized by one peak at 0.87 and a tail on the left side (or small-FRET population), consistent with the fact that the FRET signal remains high (~0.85) most of the time in such traces (Figs 3d and 5a). With the increase of the 5′-tail length, the small-FRET population increases, with a corresponding decrease of the main population under the peak (Fig. 5c), meaning that the two RecA-like domains may slide farther away from the 5′ end (Fig. 3e, state 5).

### G4 structure regulated WRN’s activity in different structural environments

**Figure 6:**
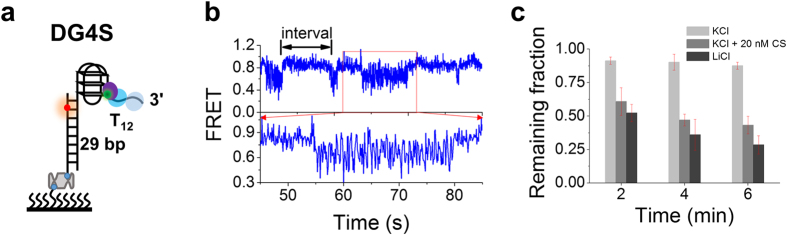
G4 hinders WRN translocation. (**a**) Schematic diagram of the G4-containing structure DG4S. (**b**) Representative FRET trace for WRN-catalyzed unwinding of DG4S in the presence of 2 nM WRN and 1 mM ATP. (**c**) Remaining fractions of DNA molecules on the coverslip surface at different times after addition of 10 nM WRN and 1 mM ATP under various buffer conditions. Light gray, gray and dark gray indicate, respectively, imaging buffer, imaging buffer containing 20 nM CS, and imaging buffer with KCl being replaced by LiCl (Error bar = s.d.; *n* = 3).

WRN has been shown to be implicated in the telomere replication of lagging strand *in vivo*^16^ and is active in unwinding intermolecular G4 DNA *in vitro*^48^. However, the molecular mechanism was not determined. To further understand the mechanism for WRN-catalyzed G4 unfolding, firstly, we prepared a DNA substrate (DG4S) with a simple three-layered G4 structure to mimic G4 in a telomere (Fig. 6a). The substrate was constructed with an ssDNA sequence containing Cy3 attached at the junction between the 3′-tail and the G4 motif, and hybridizing via its 5′ end with a stem strand complementary to it. The stem strand was labelled with Cy5 at the 6^th^ nucleotide from the 5′ end and modified by biotin at the 3′ end. In the imaging buffer, G4 has been shown to be predominantly in the well-folded conformation^25^,^26^.

**Figure 7:**
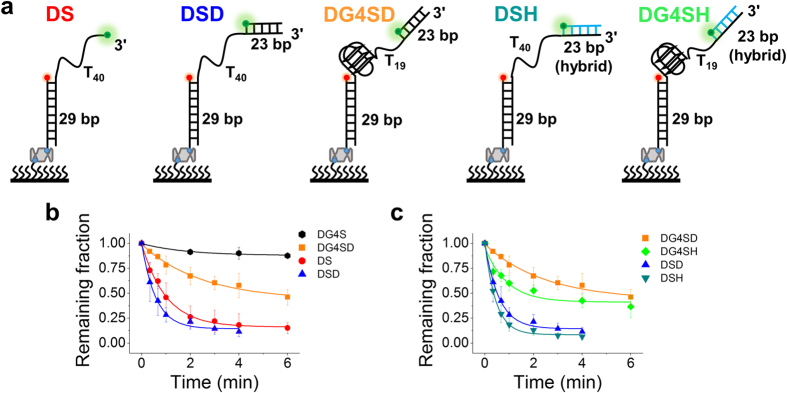
G4 hinders WRN translocation and an additional anchoring site (ss/dsDNA junction) accelerates duplex unwinding. (**a**) Schematic diagrams of substrates DS, DSD, DG4SD, DSH and DG4SH. (**b,c**) Fractions of DNA molecules remaining on the coverslip surface versus time after 10 nM WRN and 1 mM ATP were added (Error bar = s.d.; *n* = 3).

With this substrate, WRN should firstly unfold the G4 structure and then unwind the duplex. Unexpectedly, examination of the FRET curves in the presence of 2 nM WRN and 1 mM ATP (Fig. 6b) showed that complete unwinding of G4 rarely occurred, let alone complete unwinding of the duplex stem, because the FRET signal remained higher than the value, ~0.3, that corresponds to complete unfolding of the G4 structure^26^. More interestingly, intermittent FRET oscillations also appeared for this substrate (Fig. 6b), consistent with recent observations^49^. They implied that WRN might be pushed away from G4 with its rapid refolding^46^,^50^,^51^, similar to what happened with duplex DNA reannealing (Fig. 2c, state 4). Note that, during G4 unwinding, there was no displaced strand for WRN to make strand-switching, so WRN should slide back on the tracking strand, which further supports our interpretation in Fig. 2c. In addition, complete unwinding was negligible because the number of Cy5 spots did not decrease significantly even under a high protein concentration (10 nM) (Fig. 6c, light gray column). This result makes sense because G4 is thermodynamically more stable than duplex DNA and the inefficient unfolding should result from hindrance of WRN translocation by the G4 structure, similar to the case for BLM^25^,^26^. To further confirm this speculation, we performed three additional experiments: i) A short G4 complementary strand (CS, sequence listed in Supplementary Table S1) was added to the reaction buffer to externally disrupt G4 refolding; ii) KCl was replaced with LiCl in the reaction buffer, the latter does not support G4 formation even in the presence of MgCl_2_^51^,^52^; iii) As a control, a normal partial duplex DNA (named as DS in Fig. 7a) was used as the substrate. In all these experiments, efficient unwinding of the downstream duplex stem was observed (Figs 6c and 7b), just as expected.

Next, we prepared a gapped substrate DG4SD (Fig. 7a), mimicking a G4 structure that is formed in a replication lagging strand (Supplementary Fig. S8a). In this substrate, G4 was linked with a 29-nt ssDNA at the 5′ side and a 42-nt ssDNA at the 3′ side. This G4-containing 92-nt ssDNA was annealed with a 23-nt ssDNA at the 3′ end, and with a 29-nt ssDNA at the 5′ end to form the dsDNA stem. Cy3 and Cy5 were labelled at the two ss/dsDNA junctions. Upon adding 10 nM WRN and 1 mM ATP, we obtained gradual unwinding of the stem dsDNA (Fig. 7b and Table 1). When we replaced G4 with poly(dT)_21_ to construct substrate DSD, the unwinding became more efficient (Fig. 7b and Table 1), meaning the presence of G4 in DG4SD also can hinder the translocation of WRN in this structural environment. In addition, comparing the results for DG4S and DG4SD or that for DS and DSD (Fig. 7b and Table 1), we concluded that an additional anchoring site may enhance unwinding of the downstream duplex.

To further study the hindering mechanism of G4 in DG4SD, we changed the relative positions of ssDNA, dsDNA, and G4 in DG4S (Fig. 6a), yielding G4SD (Supplementary Fig. S10a). With this substrate, we could not observe the looping phenomena as shown in Fig. 3c,d, but only FRET oscillations that seldomly reached ~0.87 in the presence of 2 nM WRN and 1 mM ATP (Supplementary Fig. S10b). A reasonable explanation for the observed oscillation is that before WRN unfolded the G4 structure and reeled in the 5′ end, the refolding G4 would push the two RecA-like domains (when being in weak DNA binding state) to slide back, similar to the case for DG4S (Fig. 6b) where the whole helicase was pushed back. Repetition of these processes led to the observed oscillations.

The duplex behind WRN may also be an RNA/DNA hybrid, such as when an RNA primer exists for replication (Supplementary Fig. S8b). By replacing the 23-nt ssDNA with 23-nt RNA in DSD and DG4SD, we constructed substrates DSH and DG4SH that contain a 23-bp RNA/DNA (Fig. 7a). Interestingly, the RNA/DNA hybrid also promoted downstream duplex unwinding, even a little better than the 23-bp DNA duplex (Fig. 7c and Table 1). Taken together, these data suggest that the G4 structure can regulate WRN activity in different structural environments by hindering its translocation and WRN may reciprocate along the G4 sequence, leading to the apparently limited duplex unwinding activity.

### Mechanisms for accelerated unwinding by G4 complementary strand and additional anchor site

What is the underlying mechanism for the above observations that both G4 complementary strand (CS) and additional anchor site can promote downstream duplex unwinding? For CS, it can be easily imagined that CS anneals with the unfolded G4 sequence to prevent its refolding, thus WRN can keep on translocating rather than being excluded out (Supplementary Fig. S11a, the second panel). For the additional anchor site in DG4SD, the trailing WRN might, with the help of the ss/dsDNA junction, act as a brake to prevent slipping of the leading molecule and thus prevent G4 refolding (Supplementary Fig. S11a, the third panel). To further confirm the above models and probe the function of the additional anchor site, we studied the duplex unwinding with DG4SD in the presence of 20 nM CS (Supplementary Fig. S11a, the rightmost panel). The results showed that both the unwinding fraction and unwinding rate were decreased, compared with that for DG4S in the presence of CS or for DG4SD (Supplementary Fig. S11b and Table 1). This is reasonable, because in this case the translocation of the anchored WRN inhibited complementary strand annealing, and *vice versa*, so their effects counteracted each other (Supplementary Fig. S11a, the rightmost panel).

## Discussion

WRN plays irreplaceable roles in vertebrates, but its DNA unwinding mechanism remains elusive. In this work, we studied comprehensively the helicase activity of WRN with different biological substrates at the single-molecule level. We found repetitive activities of WRN in DNA unwinding, and further revealed that they resulted from reciprocating of WRN on the same ssDNA with the following observations: i) repetitive unwinding with forked and 3′-overhanging duplex substrates (Figs 1d and 2b and Supplementary Fig. S7b); ii) Type-II FRET bursting with 5′-tailed substrate (Fig. 3d); iii) FRET oscillation with G4-containing substrate (Fig. 6b and Supplementary Fig. S10b). The apparent low processivity of WRN^20^,^30^ can be explained as reciprocating of the helicase along ssDNA, rather than its dissociation and rebinding, or its strand switching (Fig. 2c). The ‘reciprocating model’ is consistent with observations in other studies that POT1^53^ and a DNA secondary structure of the displaced strand^11^ stimulated the apparent unwinding processivity of WRN by maintaining partially unwound strands in a melted state so that there was no annealing force to push WRN back in weak DNA binding states. Note that, the repetitive DNA unwinding of another RecQ helicase (BLM core) has been attributed to strand switching^23^,^24^, thus even helicases belonging to the same family may use different mechanisms to achieve special biological activities. This is, to our knowledge, the first evidence that nonring-shaped superfamily I/II helicase can reciprocate along the same ssDNA.

Recently, plant WRN from *Arabidopsis thaliana* has been studied under applied forces^54^. It has been found that *Arabidopsis thaliana* WRN may, after strand switching, slide back along the displaced strand of a forked DNA, resulting in highly repetitive cycling of DNA unwinding and reannealing. However, just as mentioned in that paper, direct backsliding on the tracking strand was not ruled out for the helicase. Very recently, Tippana *et al*.^49^ observed similar repetitive phenomenon when characterizing the catalytic activity of human WRN with a telomeric substrate, but they did not uncover the underlying molecular mechanism.

What might be the potential significances of WRN’s repetitive movements on different substrates *in vivo*? WRN displayed repetitive activities on different DNA structures, such as forked, overhanging and G4-containing DNAs. The repetitive activities can keep dsDNA or other DNA secondary structures such as G4 and hairpin constantly in open states, and thus may assist other enzymes, such as polymerases and telomerases, in their functioning during transcription, replication, DNA repair and telomere metabolism processes. The repetitive movements mean that one helicase can be modulated and ‘concentrated’ to function many times, thus making up a smart system that is very economical and energy-saving for cells.

It should be noted, however, that although repetitive phenomena have been observed with many different helicases^23^,^40^,^41^,^42^,^47^,^49^,^51^,^54^,^55^,^56^,^57^,^58^,^59^,^60^, it is still an open question whether they actually or always occur *in vivo*. Developing new approaches to directly record the repetitive translocation/unwinding signals in living cells is needed to resolve such issues. Furthermore, as far as WRN is concerned, repetitive behaviours were observed only with an isolated WRN protein in this study, whether and how partner proteins modify the repetitive actions of WRN are still unknown. This is an interesting research subject currently under study in our laboratory.

## Methods

### Buffers

0.8% D-glucose, 1 mg/ml glucose oxidase (266600 units/g, Sigma), 0.4 mg/ml catalase (2000–5000 units/mg, Sigma) and 4 mM Trolox were added to the reaction buffer (containing 5 mM MgCl_2_, 50 mM KCl in 20 mM Tris-HCl, pH 8.0), named as imaging buffer.

### WRN expression and purification

Truncated *Gallus gallus* WRN^512–1213^ gene was cloned into pET15b, tagged with 6 × His-SUMO at its N-terminal. The expression vector was transformed into the *Escherichia coli* ER2566 cell (NEB) and cultures were performed at 37 °C until an OD_600_ of ~0.8. Expression was induced with 0.3 mM IPTG at 18 °C overnight. After centrifugation, the cell pellets were re-suspended in ten pellet volume of lysis buffer (20 mM Tris-HCl pH 7.5, 500 mM NaCl, 10 mM Imidazole and 10% glycerol (v/v)). The cell mix was lysed by three passages through a French press and centrifuged to remove cell debris. WRN^512–1213^ was purified by fast protein liquid chromatography with sequential chromatography on Ni-NTA and Heparin. The eluted protein was incubated with Ulp to cleave His-SUMO tag overnight. Then the His-SUMO tags were recaptured by Ni-NTA. The final purified protein was dialyzed against the storage buffer (20 mM Tris-HCl pH 8.0, 300 mM NaCl, 1 mM DTT and 50% glycerol (v/v)) and flash frozen in small aliquots, then stored at −80 °C. The purity of WRN^512–1213^ protein was ≥ 90% as identified in SDS-PAGE, quantified using ImageJ (Fig. 1a, left panel). For simplicity, we used WRN to represent WRN^512–1213^ in the text, unless specified otherwise.

### DNA preparations

All oligonucleotides were ordered from Sangon Biotech (Shanghai, China). Sequences of all the oligonucleotides are listed in Supplementary Table S1. DNA substrates used in the single-molecule and the equilibrium binding measurements were annealed by incubating the mixture at 95 °C for 5 min, then followed by slow cooling down to room temperature for ~3 h. The strand with no biotin was used in excess (7:5) to reduce the probability of having non-annealed strand anchoring at the coverslip surface. The concentrations of annealed substrates were ~2.5 μM and all annealing was carried out in 100 mM KCl, 20 mM Tris-HCl (pH 8.0).

### FRET data acquisition

Single-molecule FRET study was carried out with a home-built total-internal-reflection (TIR) microscopy (objective-type) at a constant temperature (22 °C)^51^. The coverslips (Fisher Scientific, U.S.) and slides were thoroughly cleaned by a mixture of sulfuric acid and hydrogen peroxide, acetone and sodium ethoxide, then the coverslip surfaces were coated with a mixture of 99% mPEG (m-PEG-5000, Laysan Bio, Inc.) and 1% of biotin-PEG (biotin-PEG-5000, Laysan Bio, Inc.). After streptavidin at a concentration of 10 μg/ml (in 50 mM KCl, 20 mM Tris-HCl, pH 8.0) was added to the reaction chamber, 50 pM DNA was then flowed into the chamber, immobilized for 10 min, followed by washing with 10 volumes of imaging buffer to remove free DNA. WRN and ATP at different concentrations were flowed into the reaction chamber simultaneously in the imaging buffer for data acquisition with 50–100 ms time resolution, unless otherwise specified. To obtain the fraction of DNA unwound with time, a series of movies were recorded with 1.5 s duration at different times, and the acceptor (Cy5) spots were counted to represent the number of remaining DNA molecules.

### Single-molecule data analysis

The FRET efficiency was calculated using *I*_A_/(*I*_D_ + *I*_A_), where *I*_D_ and *I*_A_ are the intensity of donor and acceptor, respectively. Data analyses were carried out by scripts written in Matlab, and all data fittings were generated by Origin 8.0. Single-molecule FRET histograms were constructed by picking ~20 frames for traces of ~300 DNA molecules. The remaining fraction averaged by more than three individual experiments and the dwell time histograms from ~100 molecules were fitted with a single-exponential decay and a *γ* distribution, respectively.

### Equilibrium DNA binding assay

WRN binding to DNA was analysed by fluorescence polarization assay with Infinite F200 PRO (TECAN). Substrates (ssDNA, dsDNA) labelled with fluorescein were used in our study. Proteins of varying amount were added to a 150 μl aliquot of reaction buffer (20 mM Tris-HCl, pH 8.0, 50 mM KCl, 5 mM MgCl_2_) containing 5 nM DNA. Each time, the solution was allowed to equilibrate for five minutes at 25 °C, and then the fluorescence polarization anisotropy was measured. We obtained the equilibrium dissociation constant from fitting of the data with the Hill-equation using origin 8.0.

## Additional Information

**How to cite this article:** Wu, W.-Q. *et al*. Single-molecule studies reveal reciprocating of WRN helicase core along ssDNA during DNA unwinding. *Sci. Rep.* **7**, 43954; doi: 10.1038/srep43954 (2017).

**Publisher's note:** Springer Nature remains neutral with regard to jurisdictional claims in published maps and institutional affiliations.

## Supplementary Material

Supplementary Information

## Figures and Tables

**Table 1:** Kinetic parameters of WRN-catalyzed unwinding of different substrates at 10 nM WRN and 1 mM ATP, obtained from fittings of the data in Fig. 7b,c and Supplementary Fig. S11b (Error bar = s.e.m.).

Substrate	Time (min)	Unwinding fraction	Unwinding rate (min^−1^)	Complementary strand (CS)
DS	1.01 ± 0.07	0.83 ± 0.02	0.83 ± 0.08	—
DSD	0.58 ± 0.05	0.85 ± 0.03	1.48 ± 0.18	—
DG4S	1.83 ± 0.87	0.12 ± 0.02	0.09 ± 0.05	—
DG4SD	2.48 ± 0.43	0.56 ± 0.04	0.24 ± 0.06	—
DSH	0.46 ± 0.03	0.91 ± 0.02	1.99 ± 0.17	—
DG4SH	0.90 ± 0.23	0.55 ± 0.06	0.67 ± 0.24	—
DG4S	1.83 ± 0.06	0.59 ± 0.01	0.32 ± 0.02	20 nM
DG4SD	0.97 ± 0.20	0.18 ± 0.02	0.20 ± 0.06	20 nM
