# Supplementary material for: Single-molecule studies reveal reciprocating of WRN helicase core along ssDNA during DNA unwinding
Source: Sci Rep. 2017 Mar 7;7:43954. doi: 10.1038/srep43954 (PMC5339710; doi:10.1038/srep43954)
Supplement: Supplementary Information [file srep43954-s1.pdf]

Supplementary Information

Single-molecule studies reveal reciprocating of WRN helicase core along ssDNA during DNA unwinding

Wen-Qiang Wu[1], Xi-Miao Hou[1], Bo Zhang[1], Philippe Fossé[2], Brigitte René[2], Olivier Mauffret[2], Ming Li[3,4], Shuo-Xing Dou[3,4*] and Xu-Guang Xi[1,2,*]

[1]College of Life Sciences, Northwest A&F University, Yangling 712100, China;

[2]LBPA, IDA, ENS Cachan, CNRS, Université Paris-Saclay, Cachan F-94235, France;

[3]Beijing National Laboratory for Condensed Matter Physics and CAS Key Laboratory of Soft Matter Physics, Institute of Physics, Chinese Academy of Sciences, Beijing 100190, China;

[4]School of Physical Sciences, University of Chinese Academy of Sciences, Beijing 100049, China.

*To whom correspondence should be addressed to X.-G.X. (Email: xxi01@ens-cachan.fr) or S.-X.D. (E-mail: sxdou@iphy.ac.cn)

Table S1. Structures and sequences of substrates used in the experiments.

	Structure	Sequences (5'-3') of substrates for single-molecule FRET
**Fork-17bp**	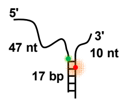	**biotin**CCTTCCTTGTCATACATTAAATATATT<br>T_{37}TTATATAAATATGTATGACAAGGAAGG
**47nt-17bp**	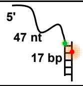	**biotin**CCTTCCTTGTCATACAT<br>T_{37}TTATATAAATATGTATGACAAGGAAGG
**Fork-29bp**	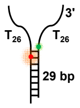	GCGTGGCACCGGTAATAGGAAATAGGAGATT$_{25}$<br>T_{26}TCTCCTATTTCCTATTACCGGTGCCACGC**biotin**
**29bp-26nt**	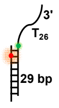	GCGTGGCACCGGTAATAGGAAATAGGAGATT$_{25}$<br>TCTCCTATTTCCTATTACCGGTGCCACGC**biotin**
**(dT)$_N$**	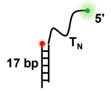	TT$_{(N-1)}$ATGTATGACAAGGAAGG<br>**biotin**CCTTCCTTGTCATACAT
**DG4S**	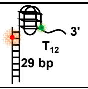	GCGTGGCACCGGTAATAGGAAATAGGAGA**GGGTTAGGGTTAGGGTTAGGG**TTTTTTTTTTTT<br>TCTCCTATTTCCTATTACCGGTGCCACGC**biotin**
**DS**	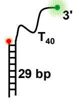	GCGTGGCACCGGTAATAGGAAATAGGAGAT$_{39}$T<br>TCTCCTATTTCCTATTACCGGTGCCACGC**biotin**
**DSD**	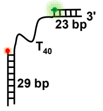	GCGTGGCACCGGTAATAGGAAATAGGAGAT$_{40}$ACACCAAGAAGTTAGACATCCGT<br>TCTCCTATTTCCTATTACCGGTGCCACGC**biotin**<br>ACGGATGTCTAACTTCTTGGTGT
**DG4SD**	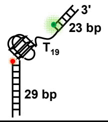	GCGTGGCACCGGTAATAGGAAATAGGAGA**GGGTTAGGGTTAGGGTTAGGG**T$_{19}$ACACCAAGAAGTTAGACATCCGT<br>TCTCCTATTTCCTATTACCGGTGCCACGC**biotin**<br>ACGGATGTCTAACTTCTTGGTGT
**DSH**	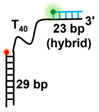	GCGTGGCACCGGTAATAGGAAATAGGAGAT$_{40}$ACACCAAGAAGTTAGACATCCGT<br>TCTCCTATTTCCTATTACCGGTGCCACGC**biotin**<br>ACGGAUGUCUAACUUCUUGGUGU
**DG4SH**	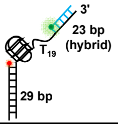	GCGTGGCACCGGTAATAGGAAATAGGAGA**GGGTTAGGGTTAGGGTTAGGG**T$_{19}$ACACCAAGAAGTTAGACATCCGT<br>TCTCCTATTTCCTATTACCGGTGCCACGC**biotin**<br>ACGGAUGUCUAACUUCUUGGUGU
**G4SD**	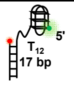	**GGGTTAGGGTTAGGGTTAGGG**T$_{12}$ATGTATGACAAGGAAGG<br>**biotin**CCTTCCTTGTCATACAT
**CS**	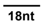	AACCCTAACCCTAACCCT
		**Sequences (5'-3') of substrates for binding**
**16nt-FAM**	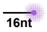	CTCTGCTCGACGGATT
**16bp-FAM**	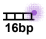	CTCTGCTCGACGGATT<br>AATCCGTCGAGCAGAG

Color label in sequences: Red, Cy5; Green, Cy3; Purple, FAM. **Bold font**: G-quarduplex sequences.

Underline/Dot underline: dsDNA or RNA/DNA hybrid forming sequences.

Table S2. Kinetic parameters for WRN-catalyzed unwinding of substrate Fork-17bp at 1 nM WRN and different ATP concentrations, obtained from fittings of the data in Fig. 1B (Error bar = s.e.m.).

[ATP]	Time (min)	Unwinding Fraction	Unwinding rate (min^{-1})
50 μM	1.39 ± 0.18	0.66 ± 0.03	0.49 ± 0.08
100 μM	0.94 ± 0.05	0.77 ± 0.01	0.81 ± 0.06
1 mM	0.51 ± 0.06	0.90 ± 0.03	1.80 ± 0.25

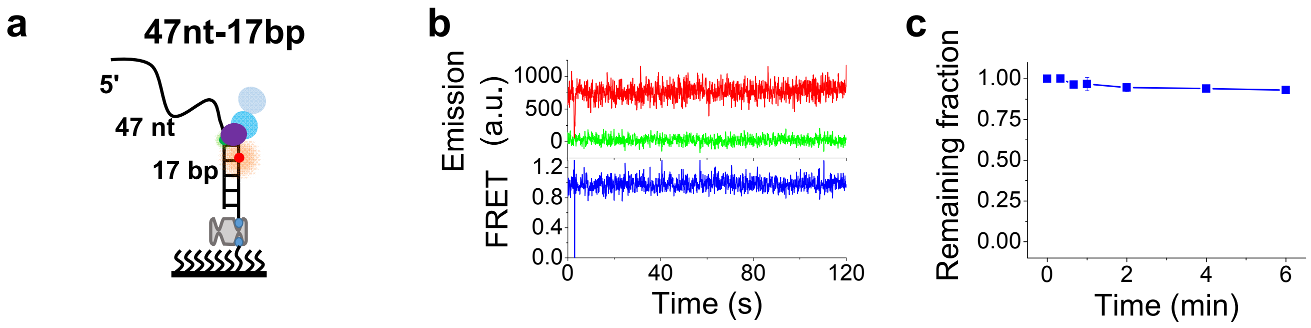

Figure S1. WRN is a strict 3'–5' helicase.

(**a**) Schematic diagram of the Cy3- and Cy5-labeled DNA construct 47nt-17bp.

(**b**) Representative fluorescence emission (upper panel) and FRET traces (lower panel) in the presence of 1 nM WRN and 1 mM ATP. No unwinding happens.

(**c**) Fraction of DNA molecules remaining on the coverslip surface versus time after addition of 1 nM WRN and 1 mM ATP. There was no obvious unwinding (Error bar = s.d.; n = 3).

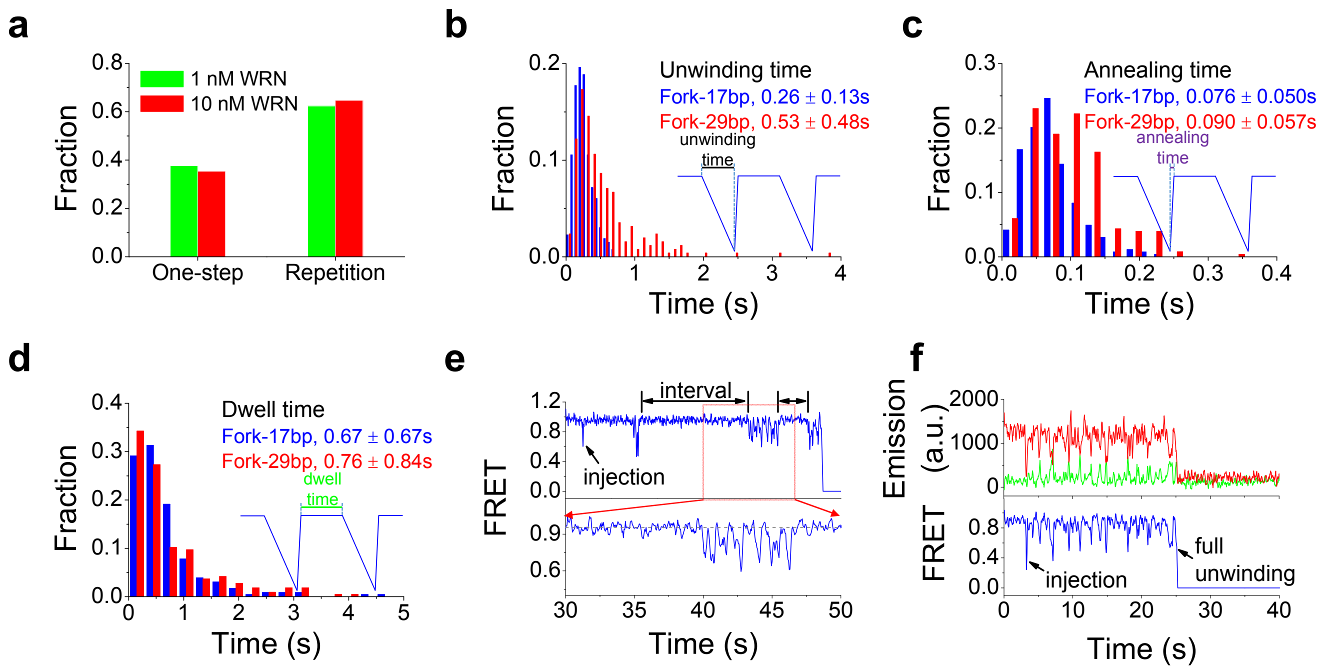

Figure S2. WRN-catalyzed unwinding of Fork-17bp and Fork-29bp.

(**a**) The ratio of one-step unwinding and repetitive unwinding was not changed at 1 nM and 10 nM WRN (one-step = 99/280 and repetition = 181/280 at 10nM WRN).

(**b-d**) Histogram of unwinding time (the transition time from high to low FRET), annealing time (the transition time from low to high FRET) and dwell time for the two substrates in the presence of 1 nM WRN and 1 mM ATP (Error = s.d.; *n* ~ 250).

(**e**) Time trace of FRET for Fork-17bp in the presence of 1 nM WRN and 1 mM ATP. The long time-intervals between two successive repetitive unfolding events indicate the probability for WRN binding is low at this protein concentration.

(**f**) Time trace of FRET for Fork-17bp when 10 nM WRN was used for preincubation and then unwinding was initiated by using 10 volumes of imaging buffer containing 1 mM ATP.

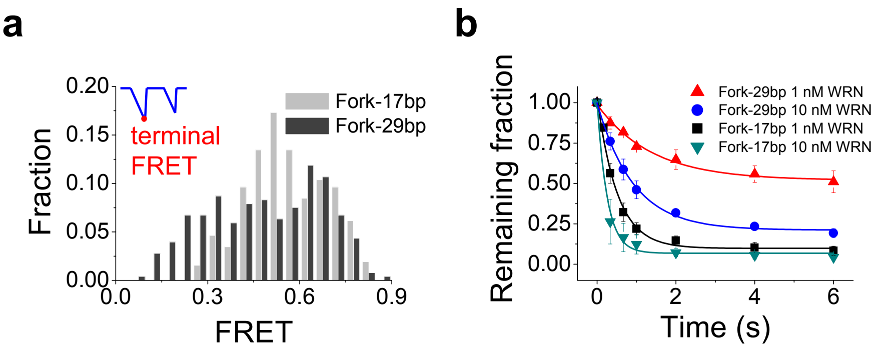

Figure S3. Differences between Fork-17bp and Fork-29bp.

(**a**) The distributions of the lowest FRET values are wide for both Fork-17bp and Fork-29bp. The concentrations of WRN and ATP are 1 nM and 1 mM, respectively.

(**b**) Remaining fraction of DNA on the coverslip surface versus time in the presence of 1 mM ATP and different concentration of WRN (Error bar = s.d.; *n* = 3).

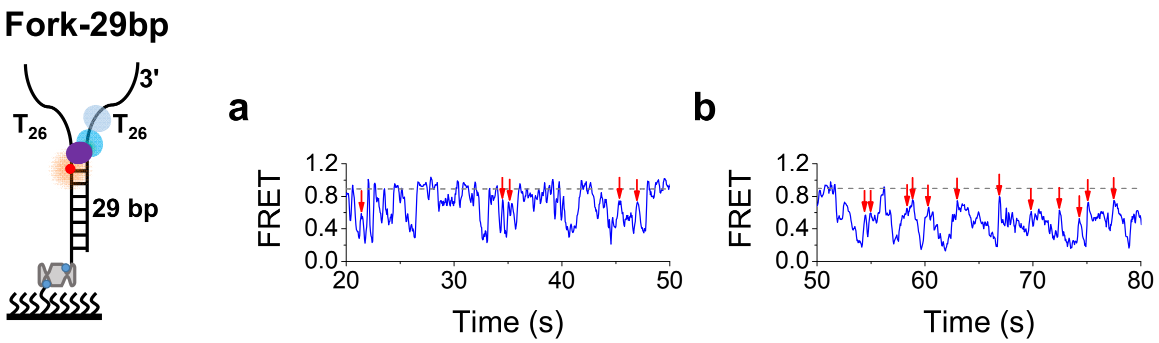

Figure S4. Selected traces of WRN-catalyzed unwinding of Fork-29bp, where FRET sometimes does not come back to its original level.

These phenomena may result from re-griping and unwinding resumption after the nucleotide state of WRN changes.

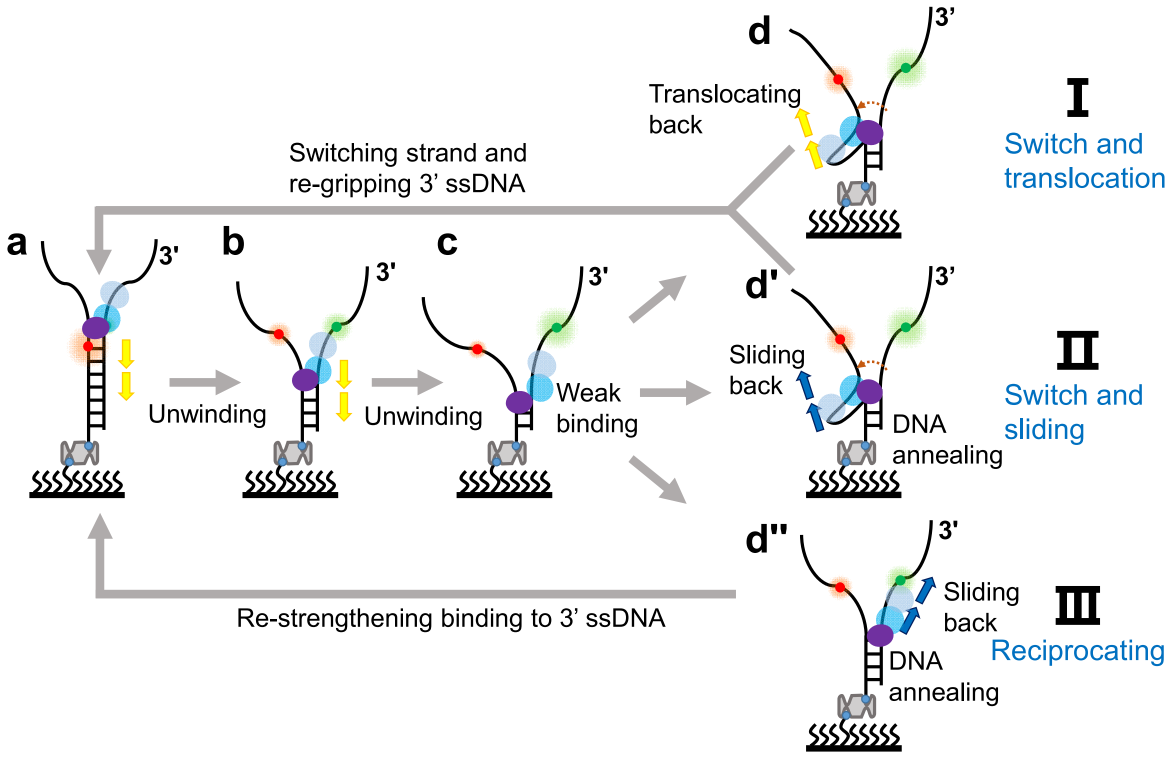

Figure S5. Three possible scenarios for the repetitive unwinding events for Fork-29bp.

(**a**) WRN binds at the ss/dsDNA junction.

(**b**) The two RecA-like domains translocate on the 3' ssDNA while RQC interacts with and melts the duplex DNA at the ss/dsDNA junction.

(**c**) In certain nucleotide state(s), the two RecA-like domains loosen their strong binding to the 3' ssDNA. From this stage, three different reaction pathways could be proposed by basing on structural information.

(**d, d'**) The two RecA-like domains of WRN may release the 3'-tail and make strand switching to the 5' tail with RQC remaining in contact with the ss/dsDNA junction. Then WRN translocatse back along the 5' tail actively (d) or be pushed back passively by DNA annealing (d'). The dashed arrows indicate strand switching of WRN from 3' to 5' ssDNA. Note that, because of the binding polarity of WRN with ssDNA, WRN could be intertwined by the 5' ssDNA.

(**d''**) WRN is pushed backwards on the tracking strand by the retreating ss/dsDNA junction due to DNA annealing.

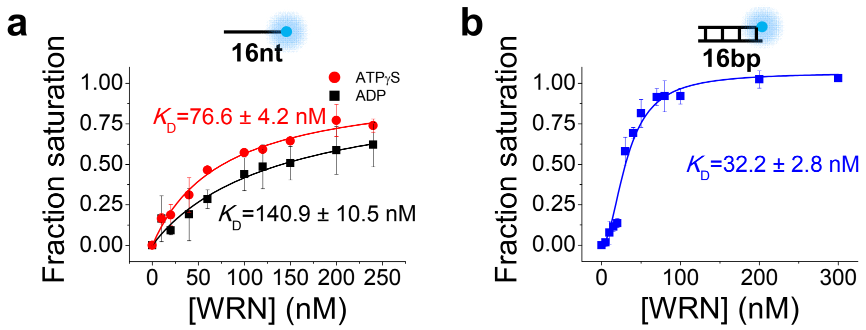

Figure S6. Determination of the binding affinities of WRN for ss- and dsDNA under equilibrium conditions.

The DNA binding dissociation constants were determined by a polarization anisotropy assay under equilibrium conditions, as described in the main context. The dissociation constants were obtained from fittings of the data using the Hill equation, $y = [WRN]^n / (K_D^n + [WRN]^n)$, where y represents the binding fraction (Error bar = s.d.; $n \geq 2$). (a) Equilibrium binding for 16nt-FAM with K_D of 76.6 ± 4.2 nM (ATPγS state, 0.2 mM) and 140.9 ± 10.5 nM (ADP state, 0.2 mM), and a Hill coefficient of 1. (b) Equilibrium binding for 16bp-FAM with K_D of 32.2 ± 2.8 nM, and a Hill coefficient of 2, the latter is expected because WRN can bind at both ends of a duplex DNA. The sequences of the DNA substrates used are given in Supplementary Table S1.

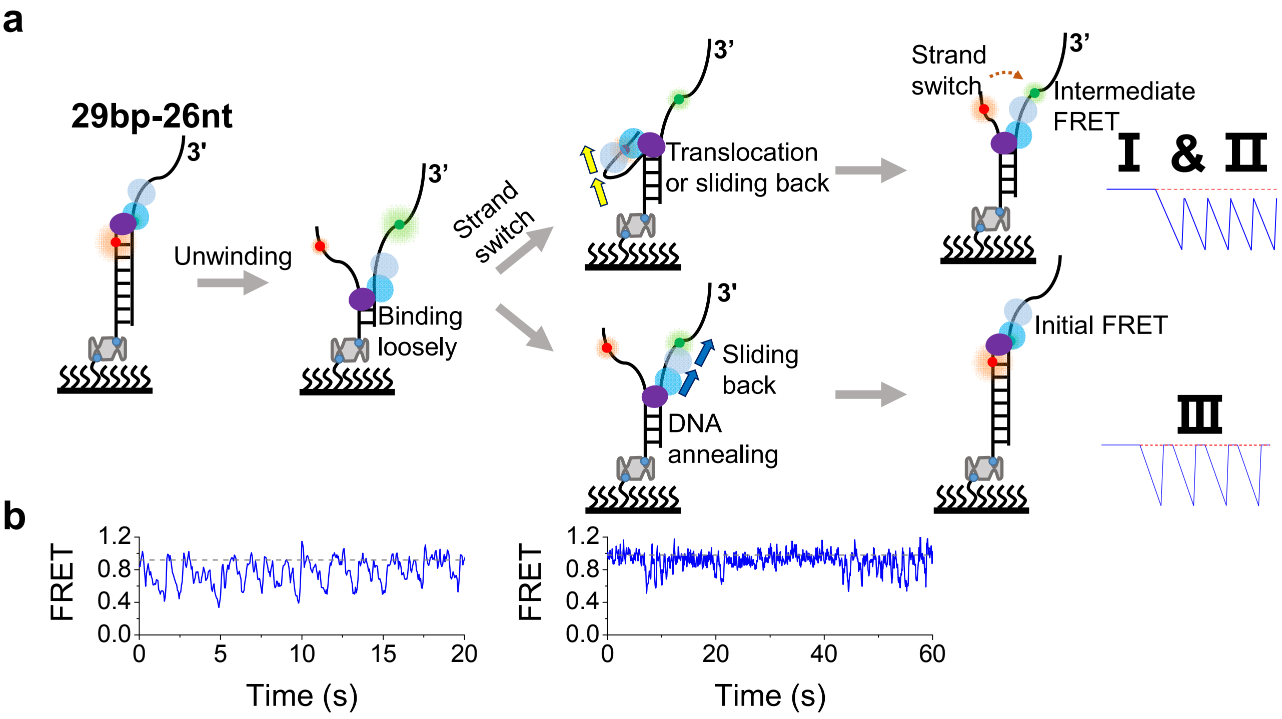

Figure S7. WRN-catalyzed repetitive unwinding of 29bp-26nt.

(**a**) The FRET signal cannot return back to its initial level for Models I and II (supplementary Fig. S5) during repetitive unwinding.

(**b**) Representative FRET traces with asymmetrical repetitive bursts characterized by a regular FRET decrease and an abrupt rising to the initial level.

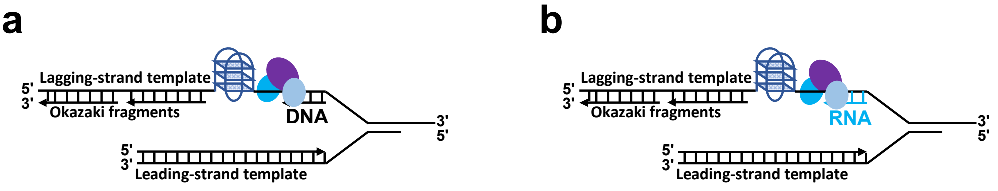

Figure S8. Schematic presentations of G4 formed in a telomere lagging-strand or in a stalled replication fork.

The junction behind WRN can be dsDNA (a) or RNA/DNA hybrid (b).

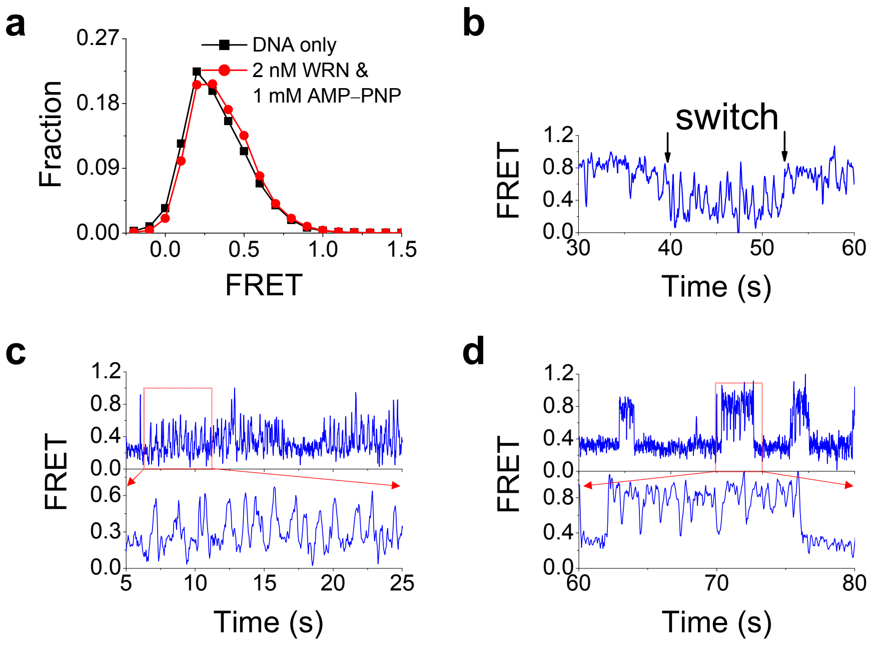

Figure S9. WRN induces looping of 5'-ssDNA overhang.

(**a**) There was no obvious FRET change for $(dT)_{40}$ in the presence of 2 nM WRN and 1 mM AMP-PNP.

(**b**) The regular sawtooth-shaped bursting and irregular bursting events could switch directly from one to another.

(**c, d**) For both types of bursting events, there exist long intervals between two successive bursting events, indicating that one enzyme is responsible for the unwinding repetitions in one bursting event.

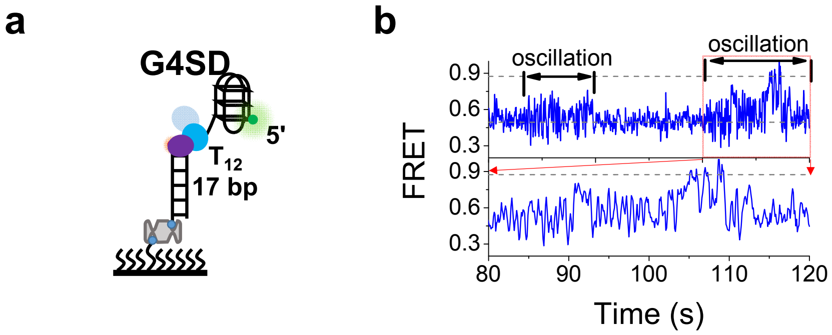

Figure S10. FRET oscillation of WRN-catalyzed unwinding of G4SD in the presence of 2 nM WRN and 1 mM ATP.

(**a**) Structure of G4SD.

(**b**) FRET oscillation with FRET seldomly reaching values (~0.87) corresponding to complete G4 unfolding.

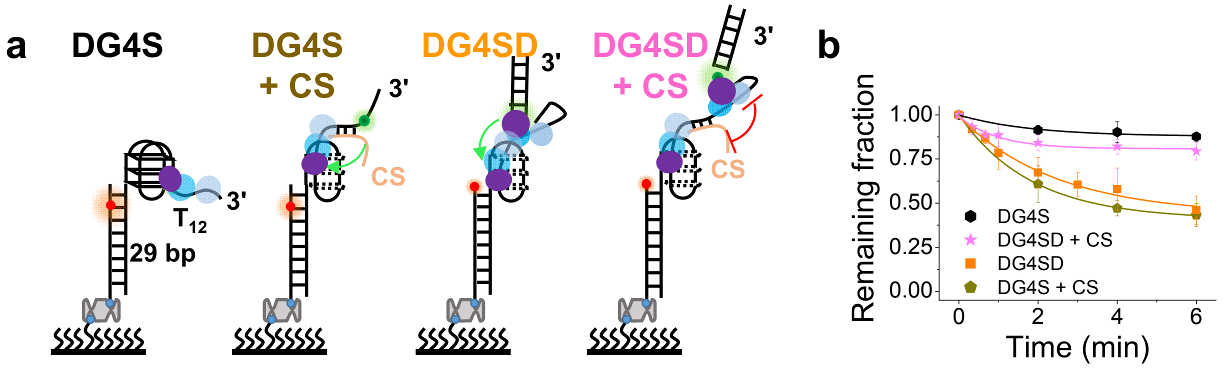

Figure S11. Mechanism of accelerated unwinding by G4 complementary strand and additional anchor site.

(**a**) Schematic diagrams of the related G4-containing substrates. The green arrow and the red bar represent activation and inhibition, respectively.

(**b**) Fractions of DNA molecules remaining on the coverslip surface versus time after addition of 10 nM WRN and 1 mM ATP (Error bar = s.d.; $n = 3$).
